# The Tibetan Antelope Population Depends on Lakes on the Tibetan Plateau

**DOI:** 10.3390/ani13233614

**Published:** 2023-11-22

**Authors:** Li Zhang, Lingyan Yan, Xiaojun Kou, Zhiyun Ouyang

**Affiliations:** 1 State Key Laboratory of Urban and Regional Ecology, Research Center for Eco-Environmental Sciences, Chinese Academy of Sciences, Beijing 100085, China; lizhang@rcees.ac.cn (L.Z.); yanlingyan10@163.com (L.Y.); 2 College of Life Sciences, Beijing Normal University, Beijing 100875, China; xj_kou@bnu.edu.cn

**Keywords:** Tibetan antelope, Tibetan Plateau, climate change, lakes

## Abstract

**Simple Summary:**

Freshwater ecosystems are embedded in terrestrial ecosystems to form an integral whole. In the context of global climate change, freshwater ecosystems may be irreversibly negatively affected; thus, aquatic organisms dependent on freshwater ecosystems are severely affected. However, terrestrial life may also experience unexpected crises due to the collapse of freshwater ecosystems, and we know very little about the relationship between terrestrial life and freshwater ecosystems. Therefore, we conducted our research on the Tibetan Plateau, an area with a dense distribution of lakes. We analyzed the relationship between the number of Tibetan antelopes (an ungulate mammal distributed in the region) and the number of lakes, and the distribution of the terrain and vegetation around the lake. Our results indicated that a greater abundance of Tibetan antelopes was associated with larger lakes, flatter terrain, and proximity to lakes, which may all be factors that affect the survival of this species. Our study suggests that lakes may provide a better habitat for ungulates in such a fragile ecosystem, including a different predation risk and more food resources, and this also provides a new research idea for the study of multi-species coexistence on the Tibetan Plateau.

**Abstract:**

The influence of freshwater ecosystems on terrestrial taxa in high-altitude regions with challenging access, such as the Tibetan Plateau, remains inadequately understood. This knowledge gap is particularly significant due to the fragility of these ecosystems, characterized by low primary productivity. Ungulates, in particular, may exhibit high sensitivity to even minor alterations in plant availability, potentially stemming from global climate change. Consequently, the investigation of these ecosystems may offer valuable insights into addressing future challenges posed by climate change. Here, to fill this knowledge gap, we explore the relationship between lakes and Tibetan antelopes in an even more vulnerable region, the Tibetan Plateau. We found that the Tibetan antelope population was higher in areas with larger lakes, and where the terrain near the lakes was flatter. At the same time, vegetation cover and plant diversity were higher near the lake compared to areas farther away from the lake. This phenomenon can be elucidated by the fact that lakes offer Tibetan antelopes a richer food supply and reduced predation risk. Our study provides new perspectives for researchers to explore the cross-ecosystem impacts of climate change.

## 1. Introduction

Freshwater ecosystems are embedded in terrestrial ecosystems and communicate with each other to form dynamic system functions [1]. Existing research has individually focused on studies within systems [2,3,4], such as significant changes in the frequency or intensity of heatwaves as global temperatures rise, increasing the risk of irreversible changes in lake ecosystems [5]. However, freshwater ecosystems are intricately interconnected with other environmental systems, fostering continuous interactions [6,7]. Previous research has underscored the profound influence of terrestrial vegetation on the physical and chemical characteristics of rivers. This influence is manifested through processes such as soil fixation and alterations in surface runoff patterns [7]. Although, we understand that complex energy transport processes exist between ecosystems, which provides the necessary theoretical support to understand cross-ecosystem impacts. Unfortunately, we lack necessary prior knowledge on how freshwater ecosystems impact terrestrial mammals in local terrestrial landscapes. This prevents us from setting the framework for biodiversity protection in response to the global climate change crisis.

Tibet, located in central Asia, boasts an average altitude of approximately 4000 meters above sea level [8]. This region plays a pivotal role in environmental studies due to its susceptibility to the impacts of global warming [9,10]. Notably, global warming has ushered in several significant changes across the Tibetan Plateau. For instance, it has led to an advancement in the greening period of vegetation, an elevation in the plateau’s tree line, and an expansion of its lakes [11,12,13,14,15]. These alterations have the potential to bring about lasting and irreversible transformations in the ecological functioning of the original ecosystem. Tibet’s landscape is punctuated with numerous lakes [16], which play a crucial role in providing essential water resources for both flora and fauna in this arid environment [17]. The ongoing, rapid climate change has resulted in a noticeable shift in precipitation patterns, with increased precipitation in the northwest and decreased precipitation in the south. This change has profoundly influenced the spatial distribution of lakes, introducing considerable heterogeneity to their presence across the region [18,19]. This unique scenario presents a valuable opportunity to investigate the impact of lake ecosystems on terrestrial organisms, going beyond their aquatic influence.

The Tibetan antelope (*Pantholops hodgsoni*) is a Class I endangered species in China [20]. The Tibetan antelope is a wild animal unique to the Tibetan Plateau, mainly distributed across 3700–5500 m alpine desert grassland and plateau grassland in the Qinghai, Tibet and Xinjiang provinces of China [21]. In 2016, it was listed as Near Threatened in the IUCN “Red List of Threatened Species” [21]. In recent years, with the implementation of protection policies and the strengthening of protection efforts, the population of Tibetan antelopes has gradually recovered; however, increasing human influences, such as climate change, infrastructure construction, and grazing, on the Tibetan Plateau still lead to habitat fragmentation and the loss of wild ungulates such as Tibetan antelopes, directly threatening their survival and population continuation. The Tibetan antelope is a migratory species. In June every year, female Tibetan antelopes begin to gather and migrate to the summer lambing area, and after giving birth, in August, female Tibetan antelopes return to their wintering habitat [22]. Every year, Tibetan antelopes need to spend three quarters of their time in the wintering area, which is mostly warm and humid grassland, or river and lake beach that is close to a water source, with high vegetation coverage and abundant edible plants [23]. Medium ungulates such as the Tibetan antelope are the main prey of predators such as the Tibetan brown bear (*Ursus arctos pruinosus*) and wolves (*Canis lupus*), and the stability of their populations is important for the stability of predators and the integrity of local ecosystems [24,25]. Due to temperature and precipitation restrictions, the Tibetan Plateau has a fragile ecological environment and relatively low primary productivity [26,27,28]. For animals, there is a trade-off between energy gain and risk, and the Tibetan antelope is no exception [29]. The proximity of the lake may allow higher primary productivity [30], but it may also be accompanied by an elevated risk of exposure to predators due to the flat terrain. However, overall, the effect of the lake on the species is not clear. 

Here, in order to understand the influence of lakes on the Tibetan antelope, we carried out a survey of the Tibetan antelope population in the Tibetan region, using the point transect method. We hypothesize that the larger the lake area, the higher the Tibetan antelope population, which represents the general effect of the lake on the Tibetan antelope. The closer the distance to the lake, the flatter the terrain and the better the vegetation condition, which represents the influence of the lake on the energy acquisition and predator exposure risk of the Tibetan antelope. We analyzed the relationship between the lake area and the Tibetan antelope population, and analyzed the distance-dependent model of slope and vegetation around the lake. Finally, we discussed the potential path of the lake’s impact on mammals.

## 2. Materials and Methods

### 2.1. Study Area

The average elevation of the Tibetan Plateau is over 4000 m, ranging from 26°00′ to 39°47′ N and 73°19′ to 104°47′ E, with a total area of about 3083,400 km^2^, an average annual temperature of 4.85 °C, and an average annual precipitation of 415.3 mm [31]. According to the comprehensive regional-scale remote-sensing products of land cover, the land cover in the study area is mainly grassland, accounting for about 58%; alpine vegetation is mainly distributed in the western high-altitude mountains, accounting for about 5%; forest is concentrated in the southeast region, accounting for about 8%; and other land cover types include artificial surface and shrubs [32]. It is an important ecological functional area with global significance across the whole Northern Hemisphere and Asia [31].

### 2.2. Point Transect Survey

**Figure 1:**
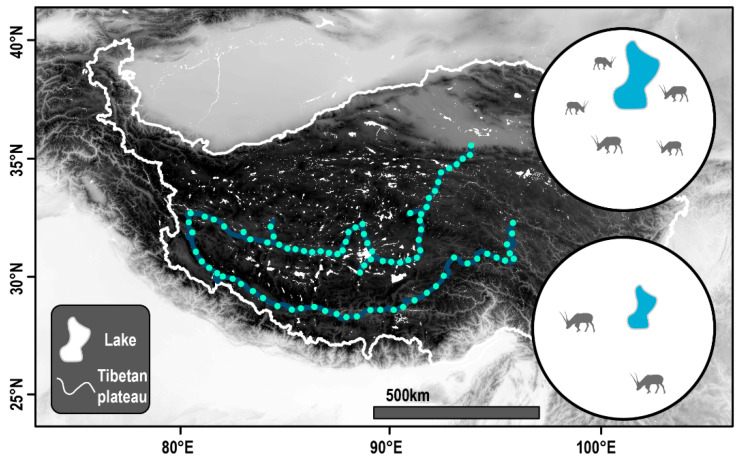
Study area map. The spatial positions of the transect and the observation point are indicated with green dot.

We conducted a point transect survey on the Tibetan Plateau from August 2020 to October 2020. We first planned the design of continuous courses to cross more diverse lake distribution areas while also ensuring the operability of the point transect survey [33]. Due to the terrain’s complexity, the target was potentially inaccessible. The courses were 5230 km long. We randomly set survey points on the course, and each survey point was more than 50 km apart, with a total of 87 survey points (Figure 1). When we arrived at a certain investigation point, we observed whether Tibetan antelopes were present, using a telescope, and then filmed the population of Tibetan antelopes in a circular area within 15 km with a digital camera and a telephoto lens with a 400 mm focal segment. The investigation lasted 20 min, and we counted the individual numbers of Tibetan antelopes in the area. GPS was used to record the latitude and longitude of the survey points.

### 2.3. Environmental Data Acquisition

After obtaining the abundance of Tibetan antelopes, we needed to obtain the corresponding environmental data, including the complete lake area intersecting the survey plots, to determine whether the lake area affects the Tibetan antelope abundance. At the same time, we also needed to obtain slope information about different lake gradients to assess the complexity of the terrain around the lake. The complexity of the terrain is related to the predation risk experienced by prey, and flatter terrain helps prey to detect predators in advance, reducing the predation risk.

We used the published land cover remote sensing product to obtain the lake layer (http://www.globallandcover.com/ (accessed on 15 October 2022)), and then analyzed the buffer zone at the observation points (Arcgis 10.5, ESRI, Inc., Redlands, CA, USA), with a buffer radius of 15 km as our survey area at each point. Then, the lake layer was clipped with the buffer, and the lake area within the range was calculated to obtain the pairing data of the number of individuals in the lake area (Arcgis 10.5, ESRI, Inc., Redlands, CA, USA).

We utilized the released topographic map (https://www.gscloud.cn (accessed on 20 October 2022)) for slope analysis to obtain the slope layer of the study area. The distance layer was generated with the lake as the center, and the value of each pixel in the layer represents the Euclidean distance from the nearest lake (Arcgis 10.5, ESRI, Inc., Redlands, CA, USA). Subsequently, 50 random points were generated in each survey area, and slope and distance information was extracted to random points to obtain distance–slope paired data in the survey area [34].

### 2.4. Vegetation Survey

For the transect survey, we conducted vegetation surveys around 20 lakes, including 10 freshwater lakes and 10 saltwater lakes. Three 10 × 10 m samples were set within different distance gradients (100 m, 500 m, 1 km, 3 km, 5 km and 10 km) from the water body to investigate vegetation coverage and plant species. When conducting distance sampling, we first observed the distribution of lakes to ensure that only one lake was sampled. If the distribution distance of multiple lakes was less than 100 m, we treated multiple lakes as one large lake. We did not sample the area surrounded by lakes to avoid the vegetation being affected by different lakes with different distance gradients.

### 2.5. Statistical Analysis

Spearman correlation analysis was carried out on the data of the lake area–individual number and distance–slope, respectively, to explore whether the data had significant positive or negative trends [35]. We used nonlinear regression to fit the logistic curve to explore the relationship between the distance from lakes and vegetation coverage and plant species, and we analyzed freshwater lakes and saltwater lakes separately. The analysis was performed using IBM SPSS 26.

## 3. Results

**Figure 2:**
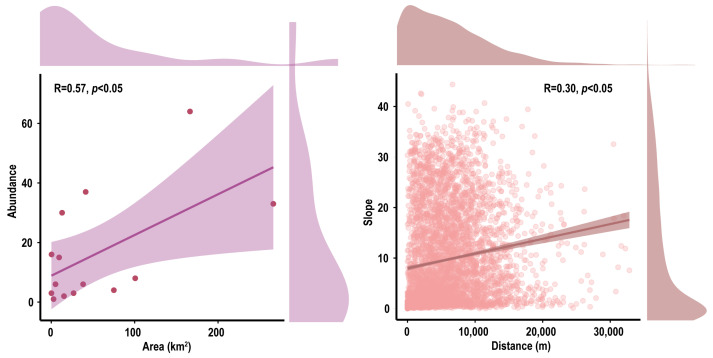
Results of paired data correlation analysis. The left side represents the relationship between lake area and the individual number of Tibetan antelopes, and the right side represents the distance dependence of the slope near the lake. The purple dots represent the Tibetan antelope sampling plots, and the brown dots represent the slope sampling plots.

A total of 228 Tibetan antelope individuals were detected, distributed across 14 sampling plots (Figures S1–S6). There was a significant positive correlation between the lake area and antelope abundance (R = 0.57, *p* < 0.05) (Figure 2) (Table S1). In all 87 areas we surveyed, the closer to the lake, the smaller the slope (R = 0.3, *p* < 0.05) (Figure 2) (Table S2).

**Figure 3:**
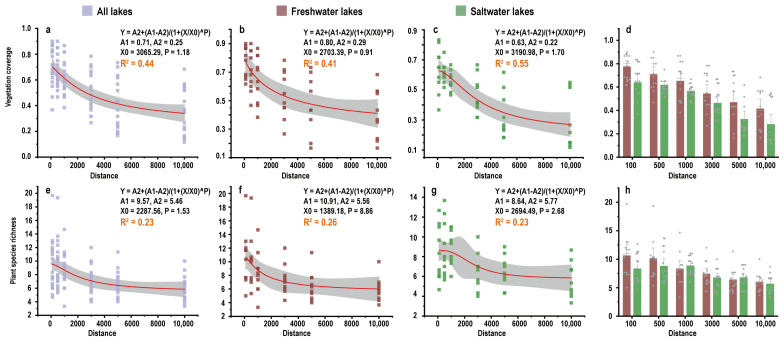
The relationship between the distance from lakes and vegetation coverage and plant species. (**a**–**c**) represents the relationship between vegetation coverage and distance of all lakes, freshwater lakes and saltwater lakes respectively, and (**d**) represents the comparison of vegetation coverage in freshwater lakes and saltwater lakes at the same distance. (**e**–**g**) represents the relationship between plant species and distance of all lakes, freshwater lakes and saltwater lakes respectively, and (**h**) represents the comparison of plant species in freshwater lakes and saltwater lakes at the same distance.

A total of 360 vegetation samples were obtained from 10 freshwater lakes and 10 saltwater lakes, and 18 samples were obtained for each lake (Table S3). In the analysis, the three samples within the distance gradient were averaged to ensure the robustness of the results. The results showed that the vegetation coverage and plant species decreased significantly with distance from the lake (R^2^ = 0.23–0.55, *p* < 0.05) (Figure 3a,e); both freshwater lakes and saltwater lakes were consistent with this trend (Figure 3b,c,f,g). However, there are some differences in the variation trends of vegetation around saltwater and freshwater lakes. The vegetation coverage of saltwater lakes is always lower than that of freshwater lakes, in different distance gradients (Figure 3d). However, saltwater lakes do not always have fewer plant species than freshwater lakes (Figure 3h).

## 4. Discussion

The Tibetan Plateau is a typical high-altitude ecosystem [36] in that it has relatively low primary productivity and places severe environmental pressure on various species [37], which may result in stronger bottom-up control [36]. Lakes, as widely distributed habitats on the Tibetan Plateau, may have more potential to provide high primary productivity due to their higher water conservation capacity. Exploring the relationship between lakes and mammals may provide new insights into patterns of species coexistence at high altitudes [38].

We observed a significant correlation between Tibetan antelope populations and environmental factors primarily associated with lakes. However, it is important to note that we only encountered Tibetan antelopes at 14 sampling sites, which may be considered a limited sample size and could introduce uncertainties when generalizing the study’s findings to a broader spatial context. Therefore, we recommend implementing a spatial distribution modeling approach for Tibetan antelopes in future investigations, leveraging systematic camera trap surveys [34]. This approach would allow researchers to gain a deeper understanding of the universality and spatial patterns governing the influence of lakes on Tibetan antelopes.

Our results show that the larger the lake area, the higher the abundance of the Tibetan antelope, and the flatter the terrain near the lake tends to be. This may be due to the higher soil moisture content near lakes. Our results show that the vegetation coverage and plant species near the lake are higher than those away from the lake (Figure 3). Although there are many saltwater lakes in Tibet, the surrounding plants have developed saline-alkali tolerance [39], which may provide better habitats for Tibetan antelopes and other herbivores [37]. In our study, the vegetation coverage around saltwater lakes was lower than that of freshwater lakes within the same distance gradient, but the species richness was not. Species richness and distance from the lake have a certain unimodal distribution potential. With the increase in distance, species richness first increases and then decreases, and it exceeds the freshwater lake in some distance gradients. This may be related to the salinity distribution around the lake. At the same time, we do not rule out that this may be because the gentle terrain reduces the concealing effect of micro-topography on predators [40,41]. Thus, the possibility of prey species to detect predators is increased, which has a negative impact on predators and promotes prey selection preference [29]. Based on the ambush habitat hypothesis, predators are more likely to choose a habitat with a complex structure to improve their concealment, rather than consistently choose a habitat with more abundant prey [29]. Better vegetation cover for prey also provides a better ambush landscape for predators, and Tibetan antelopes may experience higher predation risks in order to obtain good access to food. To determine the reasons for Tibetan antelopes’ preference for lakes, we believe that it is necessary to explore how vegetation and terrain around lakes influences predation risk.

Although it is not possible to determine which systemic action is more profound in shaping the relationship between the Tibetan antelopes and lakes, overall, the presence of lakes seems to increase the resource supply for antelopes, and, due to the topography, reduce the predation pressure on the antelopes. This may be universal for other medium-sized herbivores. This means that the role of lakes in shaping local biodiversity may be overlooked in a region with low primary productivity such as the Tibetan Plateau, not only for aquatic organisms but also for terrestrial mammals.

## 5. Conclusions

Our research reveals the positive influence of lakes on Tibetan antelopes within the Tibetan Plateau. Lakes are shown to offer enhanced food resources and reduced predation risk for Tibetan antelopes, owing to a combination of topographical and vegetative effects in their vicinity. It is worth noting that these features may also be appealing to other coexisting herbivores. To establish the universality of this phenomenon, comprehensive spatial analyses and investigations are needed. Despite its limitations, this study provides valuable information on the interactions among high-altitude ungulates, their predators, and the presence of water resources in a fragile ecosystem with limited existing knowledge. This, in turn, may facilitate the estimation of potential impacts of global warming in such rare ecosystems.

## Data Availability

The data presented in this study are available in Tables S1–S3.

## Supplementary Materials

The following supporting information can be downloaded at https://www.mdpi.com/article/10.3390/ani13233614/s1, Figure S1: Male Tibetan antelope, photographed in Changtang National Nature Reserve, Tibet; Figure S2: Female Tibetan antelope, photographed in Hoh Xil National Nature Reserve, Qinghai; Figure S3: Manasarovar in Tibet; Figure S4: Zhaling Lake in Qinghai; Figure S5: Selinco in Tibet; Figure S6: Pangong Lake in Tibet; Table S1: Relationship between individual numbers of Tibetan antelopes and lake area; Table S2: Relationship between distance and slope; Table S3: Vegetation sample data.
