# Supplementary material for: The Tibetan Antelope Population Depends on Lakes on the Tibetan Plateau
Source: Animals (Basel). 2023 Nov 22;13(23):3614. doi: 10.3390/ani13233614 (PMC10705376; doi:10.3390/ani13233614)

Figure S1. Male Tibetan antelope, photographed in Changtang National Nature Reserve, Tibet.

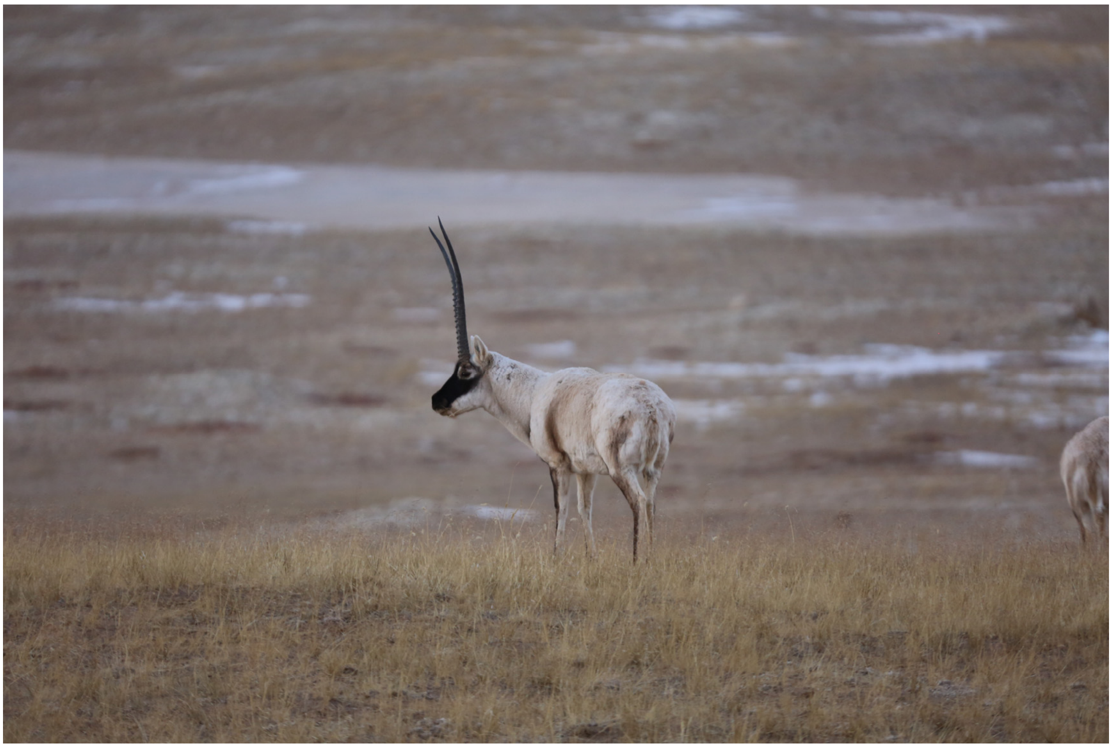

Figure S2. Female Tibetan antelope, photographed in Hoh Xil National Nature Reserve, Qinghai.

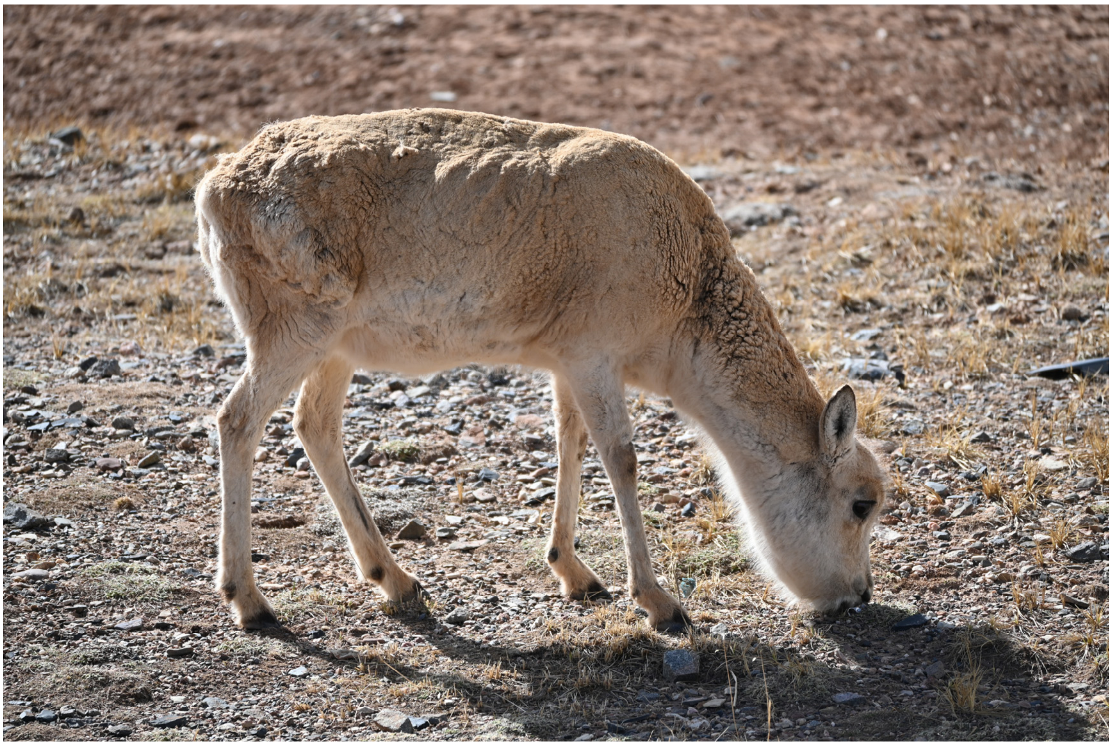

Figure S3. Manasarovar in Tibet.

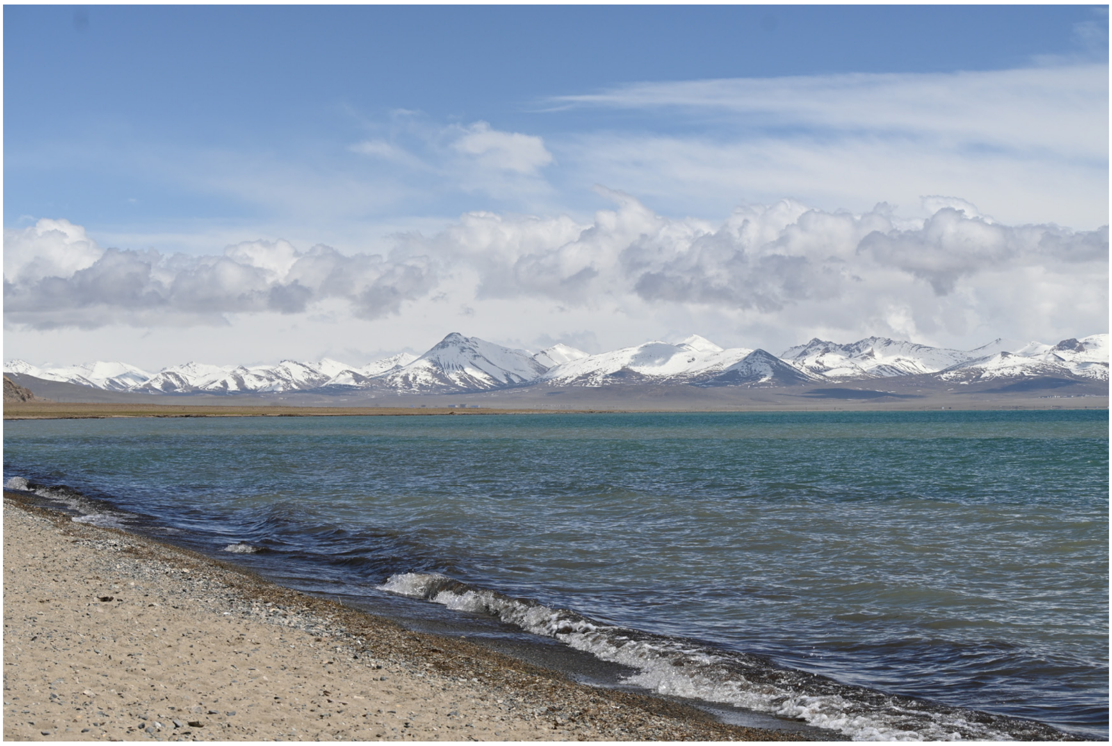

Figure S4. Zhaling Lake in Qinghai.

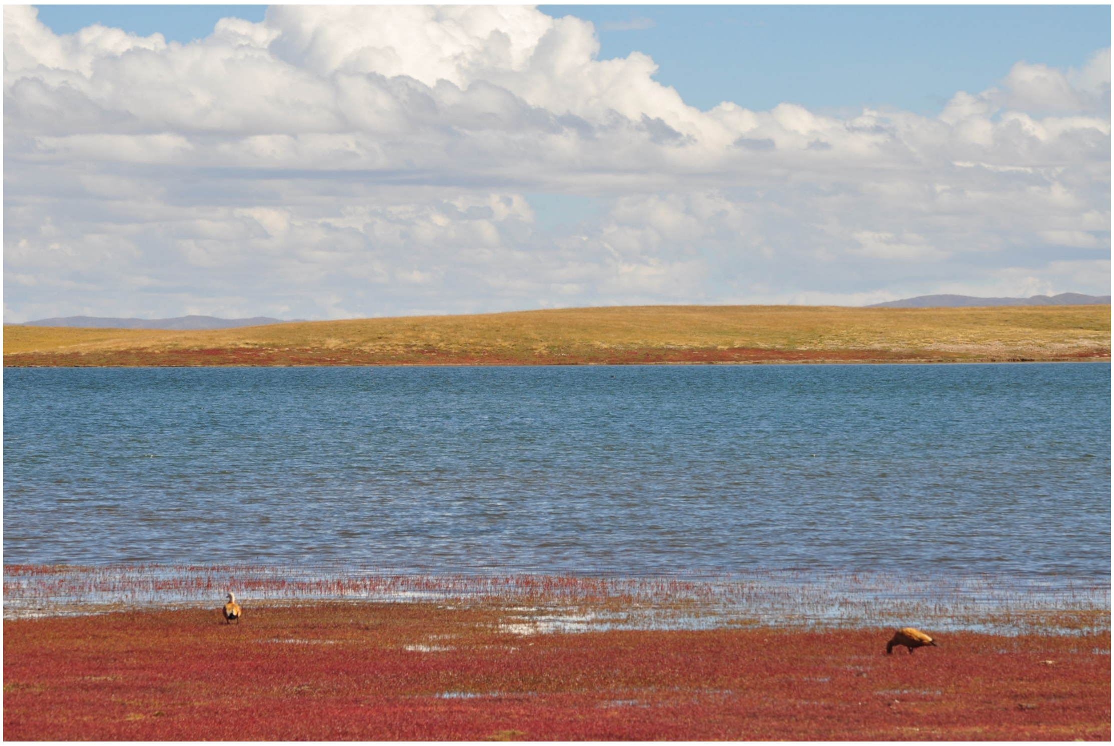

Figure S5. Selinco in Tibet.

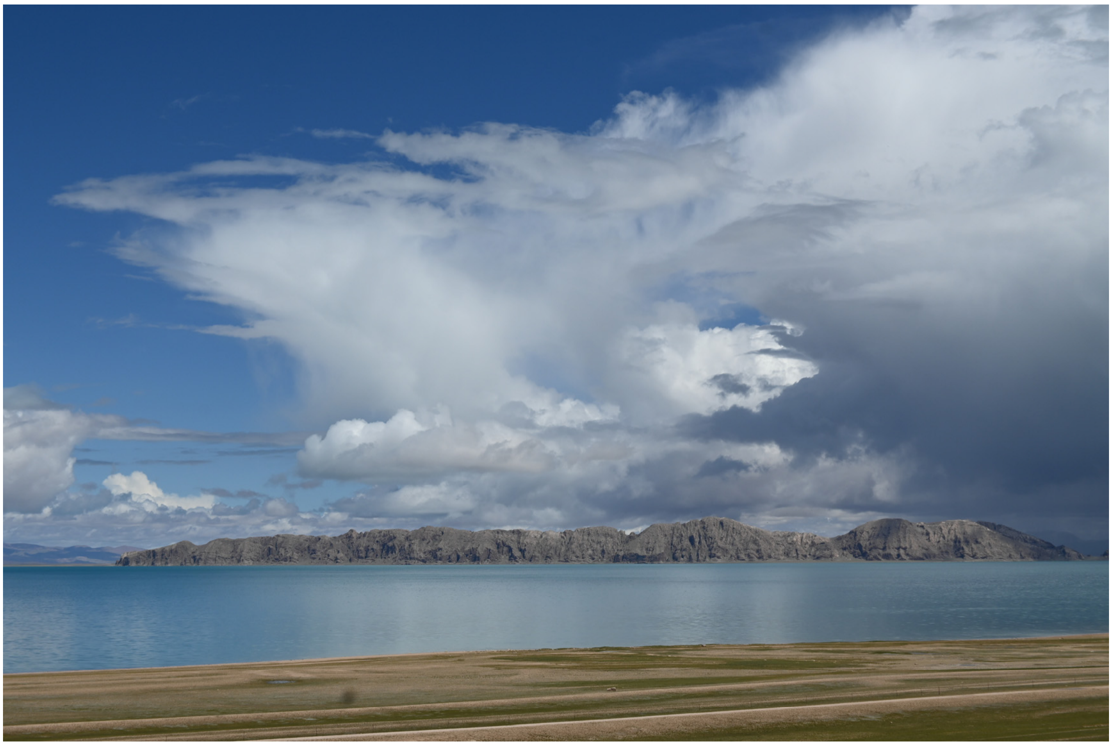

Figure S6. Pangong Lake in Tibet.

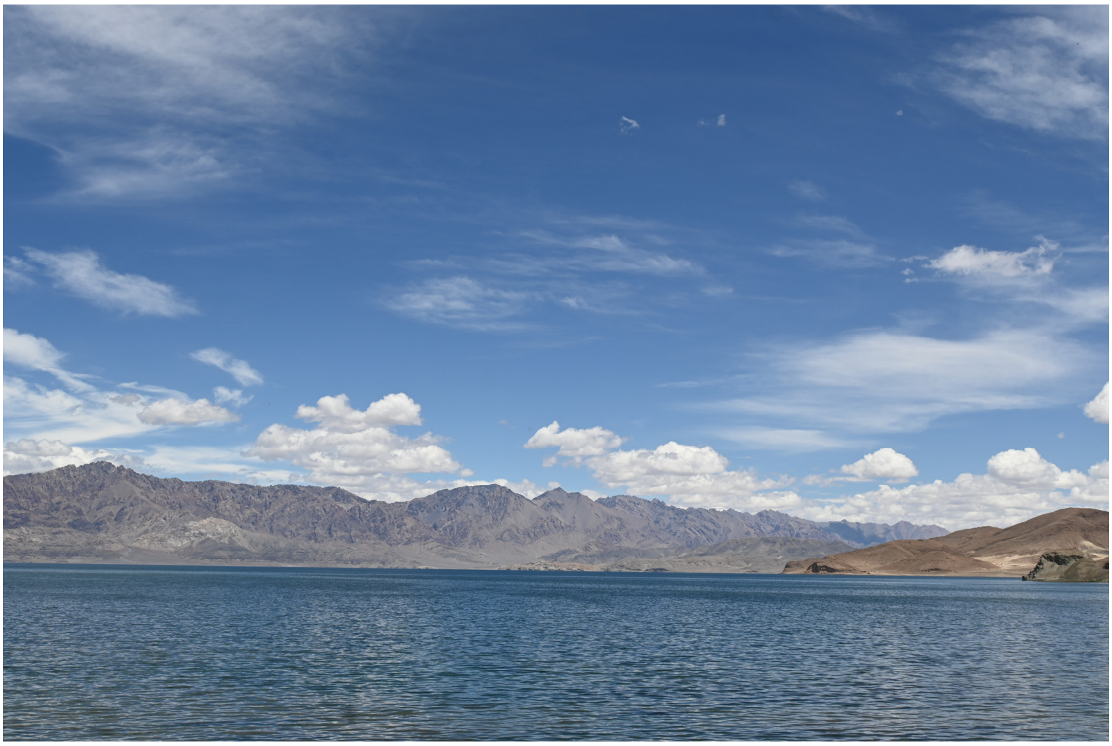

Supplement: Supplementary file 1 [file animals-13-03614-s001.zip › animals-2604328-Supplementary Figures.pdf]
